# Anti-Menopausal Effects of *Cornus officinalis* and *Ribes fasciculatum* Extract In Vitro and In Vivo

**DOI:** 10.3390/nu12020369

**Published:** 2020-01-30

**Authors:** Eunkuk Park, Eunguk Lim, Subin Yeo, Yoonjoong Yong, Junga Yang, Seon-Yong Jeong

**Affiliations:** 1 Department of Medical Genetics, Ajou University School of Medicine, Suwon 16499, Korea; jude0815@hotmail.com (E.P.); eunguk@ajou.ac.kr (E.L.); 2 Department of Biomedical Sciences, Ajou University Graduate School of Medicine, Suwon 16499, Korea; 3 Nine B Co. Ltd., Daejeon 34121, Korea; snsnans@naver.com (S.Y.); yoonjoong9b@gmail.com (Y.Y.); ajoumg@hanmail.net (J.Y.)

**Keywords:** *Cornus officinalis*, *Ribes fasciculatum*, women’s menopause, obesity, osteoporosis

## Abstract

Natural herbal medicines have been developed for the treatment and prevention of women’s menopausal symptoms. In this study, we investigated the anti-menopausal effects of *Cornus officinalis* (CO) and *Ribes fasciculatum* (RF) extracts in 3T3-L1 preadipocytes, MC3T3-E1 preosteoblasts, and COV434 granulosa cells in vitro and ovariectomized (OVX) ddY mice in vivo. Combination treatment of CO and RF extract at 7:3 ratio inhibited lipid accumulation via *Plin1* and *Adipoq* downregulation in a cocktail of dexamethasone, 3-isobutyl-1-methylxanthine, and insulin (DMI)-induced differentiated 3T3-L1 cells. In addition, CO + RF treatment significantly enhanced osteoblastic differentiation, with mineralized nodule formation occurring through the upregulation of osteoblast-inducing markers in osteoblastic MC3T3-E1 cells. Increased production of estradiol and mRNA expression of ERα (*ESR1*) were observed in androstenedione-induced COV434 granulosa cells treated with the CO + RF extract. In CO + RF-treated mice, fatty hepatocyte deposition and abdominal visceral fat tissues reduced with OVX-induced uterine atrophy. Furthermore, bone mineral density and bone mineral content were significantly enhanced by CO + RF in mouse models of ovariectomy-induced femoral bone loss. Taken together, our findings suggested that CO + RF promoted estrogenic activity and had anti-obesity and anti-osteoporotic effects in vitro and in vivo. Thus, a combination of CO and RF extracts may be a good therapeutic strategy for managing women’s menopausal syndromes.

## 1. Introduction

Menopause is a natural biological stage in women’s lives; it is characterized by the stoppage/discontinuation of menstruation due to the aging of the ovaries and usually occurs between the ages of 40 and 58 years [1,2]. During this time, the ovaries fail to produce ovarian follicles, eventually leading to an estrogen deficiency [3]. The consequent hormone imbalance leads to an increase in a number of health problems [1,2,4]. In addition, urogenital atrophy associated with estrogen deficiency may negatively affect the sex life and quality of life of postmenopausal women [5]. 

Several types of metabolic dysfunctions are attributable to a postmenopausal state due to estrogen deficiency, such as obesity, non-alcoholic fatty liver disease, heart disease, diabetes, hypertension, and an increased risk of osteoporosis and cardiovascular disease [2,6,7]. Generally, estrogen regulates insulin sensitivity of the insulin-sensitive organs, including liver, pancreas, white adipose tissue, and thermogenesis in brown adipose tissue, in addition to affecting lipid metabolism [8]. Thus, a deficiency of estrogen leads to increased visceral fat storage, an imbalance between high-density lipoprotein (HDL) and low-density lipoprotein (LDL) cholesterol levels, and increased triglyceride levels, resulting in insulin resistance [9,10]. Osteoporosis is a systemic metabolic phenomenon that frequently occurs in postmenopausal women. This skeletal disorder is characterized by structural deterioration and high fragility of bone tissue, generally caused by estrogen deficiency [6]. According to the World Health Organization (WHO), 40% of women are at risk for osteoporotic fracture worldwide and the global prevalence of osteoporosis is expected to increase considerably [11]. Estrogen maintains the balance between osteoclasts and osteoblasts in a woman’s body, thus playing a vital role in the inhibition of bone resorption and in the enhancement of bone formation [8]. Therefore, hormone supplementation therapy has been used to improve and resolve post-menopause symptoms.

However, hormone supplementation therapy has been associated with an increased risk of conditions including breast cancer, coronary heart disease, and stroke [12,13,14]. Recently, the use of natural plant-based products has increased in medical science to enhance the health of postmenopausal women without side effects [15,16,17]. However, nutraceuticals are widely used by post-menopausal women due to the limitations of standardized research protocols for menopausal complaints [18,19]. *Cornus officinalis* (CO) and *Ribes fasciculatum* (RF) are among the most common traditional medicines in East Asia. Since ancient times, both CO and RF have been used as traditional medicines due to the abundance of bioactive components, which exhibit a broad range of pharmacological activities including antidiabetic, cardioprotective, antioxidative, anti-inflammatory, anti-aging, antiallergic, neuroprotective, and antibacterial effects [20,21,22,23]. Numerous studies have suggested that a combination of natural plant extracts is more beneficial than a single extract or compound [24,25]. According to the prestigious traditional Chinese pharmacopoeia, CO has been used for more than 2000 years in the treatment of liver and kidney dysfunction as well as menopause-associated syndromes, related to endocrine system deficiency, in oriental medicine [20,21]. RF has been reported to inhibit the nuclear factor of activated T cells (NFAT) transcription factor [26] and exert a therapeutic effect via the reduction of nuclear factor-kappa B activation in macrophages in allergic inflammatory diseases [27]. A recent study of RF in *Caenorhabditis elegans* revealed that RF promoted anti-aging effects by increasing the lifespan and stress resistance of the organism [28].

The principal objective of this study was to investigate whether treatment with CO + RF improved menopausal symptoms such as estrogenic activity and had anti-obesity and anti-osteoporotic effects in vitro and in vivo. 

## 2. Results

### 2.1. Effects of Cornus officinalis (CO) + Ribes fasciculatum (RF) Extract Mixture on Lipid Metabolism

**Figure 1:**
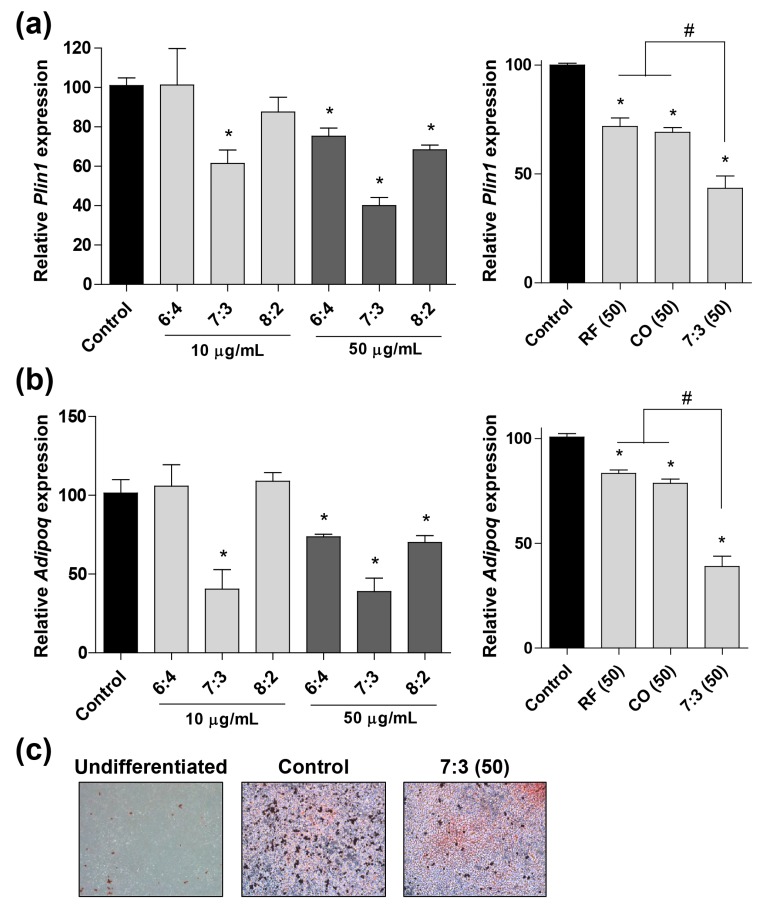
Effects of *Cornus officinalis* (CO) and *Ribes fasciculatum* (RF) extract on mRNA expression of adipogenesis-related genes and the lipid accumulation in 3T3-L1 cells. The cocktail of dexamethasone, 3-isobutyl-1-methylxanthine, and insulin (DMI)-induced 3T3-L1 cells were treated with combined CO and RF of different ratios (6:4, 7:3, or 8:2) and single extracts of CO or RF, and the mRNA expression level of adipogenesis-inducing genes, including *Plin1* (**A**) and *Adipoq* (**B**), was calculated quantitatively by qRT-PCR. (**C**) Lipid accumulation was assessed by oil red O staining and visualized under a microscope. *: *p* < 0.05 vs. control, #: *p* < 0.05 vs. 7:3 (50).

It is well known that estrogen plays a vital role in lipid metabolism in postmenopausal women. Recently, several studies demonstrated that a deficiency of estrogen promoted weight gain by causing lipid accumulation in menopausal women [7,29]. To investigate the inhibitory effect of CO + RF extract on adipogenesis, we determined the mRNA expression of key transcriptional regulators of adipogenesis-inducing genes, such as perilipin1 (*Plin1*) and adiponectin (*Adipoq*). Cells were cultured with various ratios of CO and RF (6:4, 7:3, and 8:2) and mRNA expressions of *Plin1* and *Adipoq* were measured using qRT-PCR. Neither the individual extracts nor the combination of the extracts had any cytotoxic effects in 3T3-L1 cells (Supplementary Figure S1). Differentiated 3T3-L1 cells showed high *Plin1* and *Adipoq* gene expression levels. In contrast, treatment with the CO + RF mixture (7:3 ratio) markedly decreased *Plin1* and *Adipoq* mRNA expression, compared to the single extracts of CO or RF at 50 µg/mL (Figure 1a,b). 

To further confirm the therapeutic effect of the CO + RF on lipid accumulation, we evaluated the anti-obesity effects of CO + RF on adipocyte differentiation in 3T3-L1 cells. Preadipocytic 3T3-L1 cells were treated with 3-isobutyl-1-methylxanthine, dexamethasone, and insulin (MDI) for adipocyte differentiation, and lipid accumulation was analyzed by oil red O staining. Treatment of differentiated 3T3-L1 cells with the CO + RF mixture decreased oil red O-positive cells, compared to a cocktail of dexamethasone, 3-isobutyl-1-methylxanthine, and insulin (DMI) treatment alone (Figure 1c), indicating the attenuation of lipid droplet accumulation. These results suggested that CO + RF mixture at a 7:3 ratio inhibited adipocyte differentiation through the downregulation of adipogenesis-inducing genes. 

### 2.2. Effects of CO + RF Extract Mixture on Osteoblast Differentiation

**Figure 2:**
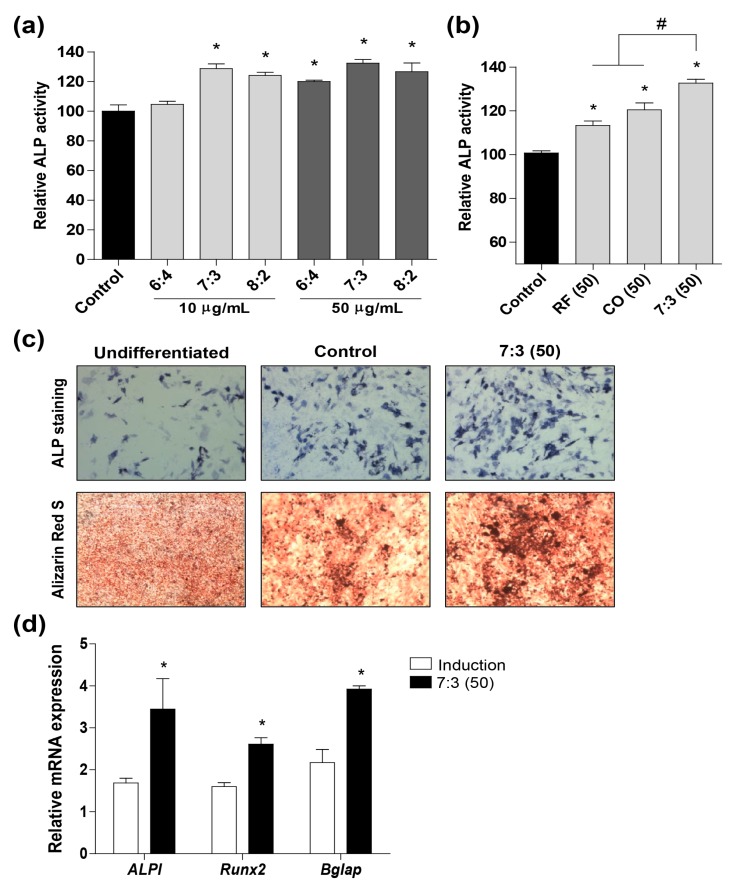
Effects of the CO + RF extract on osteoblast differentiation and mRNA expression of bone remodeling markers in preosteoblastic MC3T3-E1 cells. (**A** and **B**) Cells were treated with CO or RF extract or their combination (6:4, 7:3, and 8:2 ratios). After induction of osteoblast differentiation, the alkaline phosphatase (ALP) activity was assessed. (**C**) Osteoblast differentiation and mineralized nodule formation were assessed using the ALP assay and by Alizarin Red S staining after treatment with the CO and RF extracts in combination at a 7:3 ratio (50 μg/mL). (**D**) The relative mRNA levels of *Alpl, Runx2*, and *Bglap* in the combined CO and RF extract-treated cells were analyzed by RT-PCR. *: *p* < 0.05 vs. control, # *p* < 0.05 vs. 7:3 (50).

During menopause, estrogen levels markedly reduce, leading to increased risk of osteoporotic fractures [30]. To explore the effect of CO and RF extracts on bone formation, we investigated osteoblastic differentiation using orthodox methods of alkaline phosphatase (ALP) activity and staining, and mineralization in preosteoblast MC3T3-E1 cells. Osteoblastic cells were treated with combined SC and RF of different ratios (6:4, 7:3, or 8:2) and single extracts of CO or RF and bone formation enhancing effects and osteoblast differentiation were analyzed by ALP activity. CO and RF did not have cytotoxic effects in MC3T3-E1 cells (Supplementary Figure S2). After induction of osteoblast differentiation, the highest ALP activity was observed for the 7:3 CO + RF treatment, compared to extract alone or CO + RF at ratios of 6:4 and 8:2 (Figure 2a,b). In addition, the treatment of CO + RF at a ratio of 7:3 promoted ALP-positive staining colonies and mineralized nodule formation, compared to the control in preosteoclast cells (Figure 2c). Finally, we selected the CO + RF extract at 7:3 ratio for the subsequent experiments. To further confirm the synergistic effect of CO + RF extract on the cellular differentiation of osteoblasts, we tested the mRNA expression of bone remodeling markers, including *Alpl* (alkaline phosphatase), *Runx2* (runt-related transcription factor 2) and *Bglap* (bone gamma carboxyglutamate protein). During osteoblast differentiation, CO + RF extracts enhanced mRNA expression of bone enhancing markers (*Alpl, Runx2*, and *Bglap*) (Figure 2c). These results suggested that CO + RF extracts at a 7:3 ratio promoted a synergistic bone formation effect and osteoblast differentiation via up-regulating mRNA expression levels of *Alpl, Runx2*, and *Bglap*.

### 2.3. Anti-Menopausal Effects of CO + RF in Ovariectomized (OVX) Mice

Based on the in vitro study, we investigated the anti-menopausal effects of the CO + RF mixture in OVX mice. OVX animals are a well-recognized model of postmenopausal conditions characterized by an increase in body weight and reduction of bone mass and quality [31]. Eight-week-old female ddY mice were subjected to either an ovariectomy or sham operation (Sham). The OVX mice were divided into five groups; 1) Sham, 2) OVX control, 3) OVX and treated with 75 mg/kg/day CO + RF, 4) OVX and treated with 150 mg/kg/day CO + RF, and 5) OVX and treated with 300 mg/kg/day CO + RF (*n* = 6 in each group)**.** Each concentration of the extract was mixed with feed. Food intake between the OVX- and CO + RF-treated groups did not differ (data not shown). At the end of the experiment, body weight and total fat and uterus weights were measured and right femur bone mineral density (BMD) and bone mineral content (BMC) were analyzed using an electronic scale and PIXImus small-animal densitometer. Total fat percentage was calculated as the total fat divided by the total body weight.

### 2.4. Anti-Obesity Effects of CO + RF in OVX Mice

**Figure 3:**
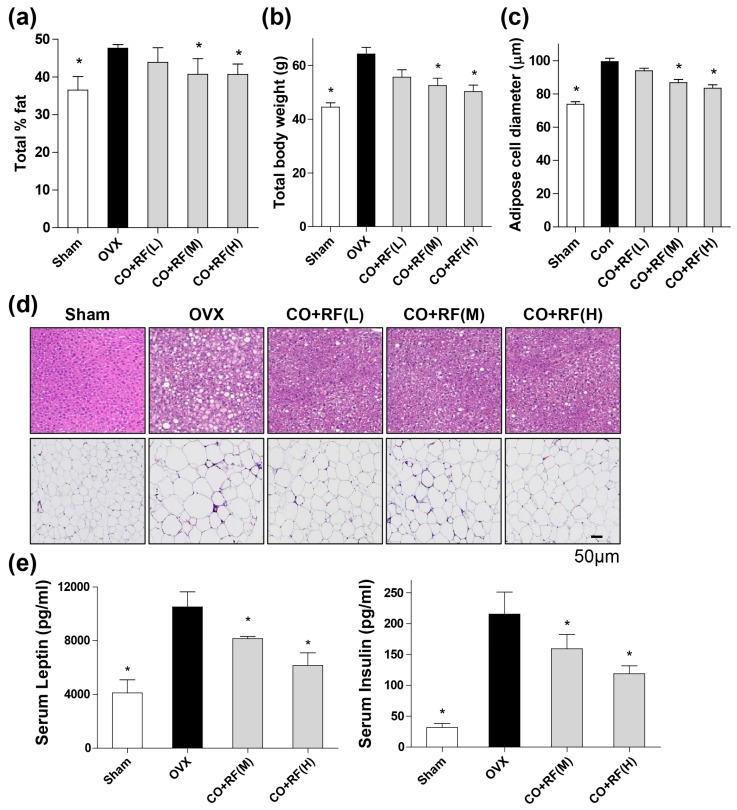
Anti-obesity effects of CO + RF in ovariectomized (OVX) mice. The OVX mice were administered with combined CO + RF extract (75 (L), 150 (M) or 300 (H) mg/kg/day) for 12 weeks. Sham: Sham operated, OVX: Non-CO + RF-administered mice. At the end of animal experiments, total % fat (**A**) and total body weight were measured (**B**) and adipose cell diameters (**C**) were measured using the CaseViewer program (3DHISTECH Ltd.). Histological images of liver and abdominal fat tissues were assessed using hematoxylin and eosin (H&E) staining (**D**). The serum levels of leptin and insulin (**E**) were measured using enzyme-linked immunosorbent assay (ELISA). *: *p* < 0.05 vs. OVX.

As expected, OVX mice showed an increase in body weight and total fat, compared to those in the sham group. However, the CO + RF-treated groups showed significantly reduced body weight and total fat percentage, compared to that in the OVX group (Figure 3a,b). Histological images of the liver and abdominal fat tissues were assessed using hematoxylin and eosin (H&E) staining. Liver tissue showed a high degree of steatosis and severe cytoplasmic vacuoles, and hepatocyte swelling and enlargement of epididymal fat tissue were observed in the OVX group. However, CO + RF treatment inhibited hepatic fatty deposition in hepatocytes and adipose cell diameter and size in adipocytes (Figure 3c,d). 

To further confirm the anti-obesity effects of CO + RF in the OVX mouse model, we examined two obesity-related hormones (leptin and insulin) from the left ventricle of mice. The blood samples were collected on the last day of experiment, serum leptin and insulin levels were measured using the enzyme-linked immunosorbent assay (ELISA). OVX-induced mice showed high levels of leptin and insulin. However, CO + RF-treated groups prevented serum concentration of leptin and insulin (Figure 3e). These results suggested that CO + RF reduced the OVX-induced weight gain by decreasing the secretion of leptin and insulin concentrations.

### 2.5. Estrogenic Effects of CO + RF on Uterus in OVX Mice and on COV434 Granulosa Cells

**Figure 4:**
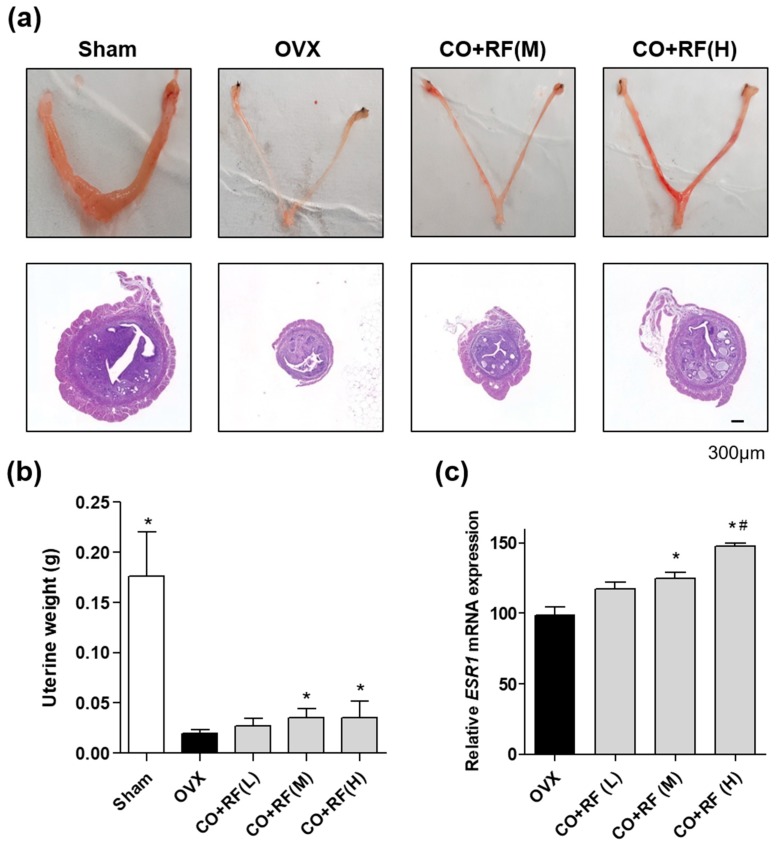
The effects of CO and RF on uterine weight and morphology and estrogen receptor *ESR1* expression in OVX mice. After CO and RF mixture treatment (75 (L), 150 (M) or 300 (H) mg/kg/day) for 12 weeks, morphological uterine atrophy was assessed by taking uterine photos and histological images of H&E staining (**A**) and measuring uterine weight (**B**). (**C**) mRNA expression of *ESR1* was measured by qRT-PCR. *: *p* < 0.05 vs. OVX, #: *p* < 0.05 vs. CO + RF (L).

Since uterine weight and morphology is regulated by estrogen concentrations [32], we investigated the effects of CO + RF mixtures of uterine weight in the OVX mice model. OVX mice resulted in reduced uterine weight and uterine atrophy, compared to the sham group. However, CO + RF at 150 and 300 mg/kg/day concentrations promoted uterine weight gain and hypertrophic changes with mRNA expression of mouse estrogen receptor alpha (*ESR1)* in mice uteruses (Figure 4a–c). 

**Figure 5:**
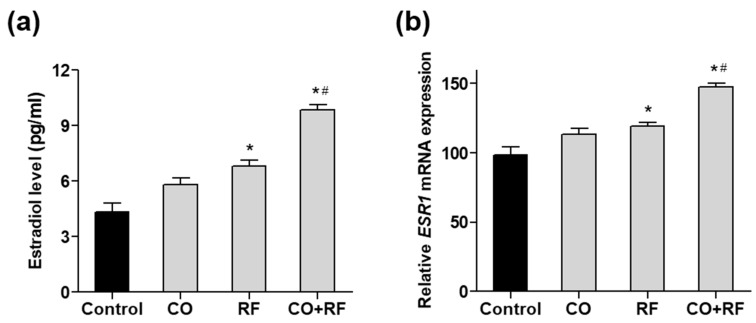
The effects of CO and RF on estradiol secretion and mRNA expression of estrogen receptor *ESR1* gene in androstenedione (ADD)-induced COV434 granulosa cells. Cells were co-treated with CO or RF extract or their combination at a 7:3 ratio (50 μg/mL) and 10 µM ADD for 48 h, and the estradiol level in the supernatant of the cell culture medium was measured by ELISA kit (**A**), and the mRNA expression level of *ESR1* gene (**B**) was calculated quantitatively by qRT-PCR. *: *p* < 0.05 vs. control, #: *p* < 0.05 vs. CO.

To further confirm the effect of CO and RF extracts on estrogen production, we evaluated estradiol synthesis induced by treatment with 10 µM androstenedione (ADD) in human ovarian COV434 granulosa cells, as previously described [33]. COV434 cells are known to express ESR1 and ESR2 and produce estradiol. We confirmed that ADD treatment significantly increased estradiol production (Supplementary Figure S3A). Treatment with the combination of CO + RF resulted in higher estradiol production than that with CO and RF single extract treatments (Figure 5a), suggesting effective estrogenic activation in COV434 granulosa cells with CO + RF treatment. Next, we evaluated the mRNA expression of human estrogen receptor alpha (*ESR1*) and beta (*ESR2*). Significantly increased *ESR1* expression was observed in the CO + RF extract-treated cells, compared with CO and RF single extract-treated cells (Figure 5b). However, the mRNA expression of *ESR2* did not differ between the groups (Supplementary Figure S3B). Similar results of the mRNA expression of *ESR1* and *ESR2* were observed in differentiated preosteoblastic MC3T3-E1 cells (Supplementary Figure S4).

### 2.6. Anti-Osteoporotic Effects of CO + RF in OVX Mice

**Figure 6:**
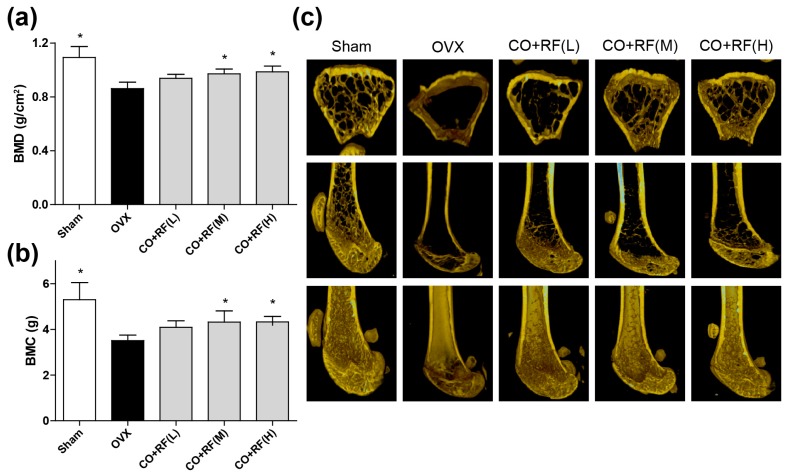
Anti-osteoporotic effects of CO + RF in OVX mice. After 12 weeks treatment, OVX-induced bone loss was evaluated by bone mineral density (BMD) (**A**) and bone mineral content (BMC) (**B**) with micro-CT image of right femur (**C**). *: *p* < 0.05 vs. OVX.

Bone mass is maintained by bone remodeling (continuation of the maturation and regeneration process of skeletal), which is regulated by two processes, bone resorption (osteoclasts) and bone formation (osteoblasts) [31,34]. However, the most unbalanced bone remodeling occurs during the menopause period, due to a lack of estrogen. To investigate whether the CO + RF mixture improves bone quality in OVX mice, we evaluated the BMD and BMC with micro-CT imaging of the right femur. OVX mice exhibited a significantly decreased femoral BMD and BMC and the micro-CT image showed trabecular bone loss compared to that in the sham group. However, animals treated with 150 and 300 mg/kg/day CO + RF showed improved BMD and BMC (Figure 6a,b) and prevention of ovariectomy-induced bone loss (Figure 6c), indicating the anti-osteoporotic effects of CO + RF. These results suggested that the CO + RF mixture may be more efficacious in reversing trabecular loss due to estrogen deficiency.

## 3. Discussion

Many therapeutic medications have been developed for the treatment of menopausal symptoms but the safety of conventional hormone replacement therapy (HRT) has been a concern for women’s health and primary care as it is associated with a high risk of undesirable side effects [8,12]. However, plant-based natural products have been used in modern times for the treatment of several diseases, with fewer adverse effects from long-term use [34,35]. Therefore, in this study, we explored the anti-menopausal effects of the CO + RF extract as an alternative anti-menopausal agent. 

Menopause is the period of ovarian estradiol and progesterone production failure with undesirable symptoms such as hot flashes, weight gain, osteoporosis, headaches, and depression. CO has been used for clinical treatment of metabolic disorders including dyslipidemia and hypoglycemia [20]. In addition, it has been reported to have protective effects in follicle depletion through a reduction in body weight and tail skin temperature in 4-vinylcyclohexene diepoxide-induced mouse model of menopause [36]. RF has been reported have anti-inflammatory effects through suppression of NFAT or nuclear factor (NF)-κB (NF-κB) activation [37]. In a recent study of RF in *C. elegans,* RF exerted anti-aging effects through increased longevity and stress resistance in the organism [24]. Numerous studies have suggested that a combination of plant extracts containing several phytochemicals results in synergistic pharmacological effects [27,28]. Despite the beneficial medical effects of CO and RF extracts, the synergistic anti-menopausal effects of the CO + RF mixture have not been studied. In this study, we evaluated the anti-obesity and anti-osteoporotic effects of CO and RF in preadipocyte 3T3-L1 and preosteoblast MC3T3-E1 cells in vitro and the OVX ddY mice model in vivo. 

Estrogen is produced from the granulosa cells, which are located in the follicles of the mammalian ovary and closely associated with the growth of oocytes, differentiation, meiosis, cytoplasmic maturation, and genomic transcriptional activity within oocytes [38,39]. The transcriptional effects of estrogen are mediated by two key estrogen receptors (ER), ER alpha (ERα), and ER beta (ERβ). The uterus is a major target female organ for reproductive hormones and consists of various cell types, including smooth muscle, stroma, glandular, and luminal epithelia. Morphological changes in the uterus are regulated by these cells in response to the concentrations of circulating estrogen and progesterone. ERα in the uterus plays an especially critical role in mediating estrogen action. Accordingly, a study showed that estrogen deficiency induced atrophy in the uterus of OVX rats [32]. In the present study, the CO + RF extract increased the mRNA expression of mouse ERα (*ESR1)* in the murine uterus and estradiol secretion in ADD-induced COV434 granulosa cells. Hence, CO + RF treatment promoted estrogenic activation and prevented ovariectomy-induced uterine atrophy by upregulating mouse ERα (*ESR1)*. 

Generally, estrogen regulates insulin sensitivity of the insulin-sensitive organs, including the liver, pancreas, and white adipose tissue, and thermogenesis in brown adipose tissue, in addition to affecting lipid metabolism [7,29]. Thus, estrogen deficiency increased triglyceride levels in insulin resistance, leading to increased visceral fat storage and imbalance between HDL and LDL cholesterol [9]. In postmenopausal women, estrogen deficiency may be an important obesity-triggering factor [40]. Adipogenic differentiation is regulated by modulators of adipocyte lipid metabolism, such as *Plin1* and *Adipoq* [41]. *Plin1* is a lipid droplet-associated gene and is known as a key regulator of lipid storage; it is involved in modulating the lipolysis of cytoplasmic triacylglycerol deposits in adipocytes [42]. *Adipoq* is a novel adipose-specific gene whose expression is markedly increased in differentiated 3T3-L1 preadipocytes [43]. A recent study demonstrated a strong association of *Plin1* and *Adipoq* genes with adipogenesis [44]. In the present study, histological visualization of oil red O staining showed that CO + RF inhibited lipid accumulation in differentiated 3T3-L1 cells. Additionally, the mRNA expression levels of *Plin1* and *Adipoq* genes were significantly downregulated in CO + RF-treated 3T3-l1 cells. These results suggested that the CO + RF mixture prevented adipocyte differentiation through the downregulation of *Plin1* and *Adipoq* expression in 3T3-L1 cells. Similarly, in an in vivo experiment, markedly reduced OVX-induced weight gain, total % fat, fatty hepatocyte deposition, and abdominal visceral fat with decreased obesity-associated hormones were observed in CO + RF-treated mice. These results suggested potential anti-obesity effects of CO + RF in the OVX mouse model. 

The highly coordinative relationship between adipogenesis and osteogenesis is involved in the maintenance of bone quality and quantity [44]. Bone remodeling is maintained by the balance of bone formation (osteoblast) and bone resorption (osteoclast) [31,34]. Osteoblasts play an important role in the maintenance of skeletal architecture and these cells secrete factors that regulate the deposition of bone matrix and osteoclast differentiation [45]. ALP activity is a key biochemical marker for bone formation and mineralization during osteoblast differentiation [46]. In addition, osteoblast differentiation is regulated by bone formation enhancing markers, such as *Alpl* (alkaline phosphatase, ALP), *Runx2* (runt-related transcription factor 2, Runx2), and *Bglap* (bone gamma carboxyglutamate protein, Osteocalcin) [47,48]. The deficiency of bone replacement during menopause causes an increase in bone mass loss, due to more bone resorption than bone formation, leading to decreased BMD and BMC [49]. In the present study, CO + RF promoted osteoblast differentiation and mineralized nodule formation via increasing mRNA expression of bone enhancing markers (*Alpl, Runx2*, and *Bglap*) in preosteoblast MC3T3-E1 cells. In addition, we evaluated the bone quality in an osteoporosis model by measuring BMD and BMC and confirmed ovariectomy-induced bone loss on micro-CT images of the right femur. Our findings showed that the treatment with the CO + RF mixture increased BMD and BMC of the right femur and prevented trabecular bone loss in estrogen deficiency-induced osteoporosis. These results suggest the anti-osteoporotic effects of the CO + RF mixture in vivo.

## 4. Materials and Methods

### 4.1. Extracts and Cell Culture 

CO and RF extracts were provided from the Korean Plant Extract Bank in the Korea Research Institute of Bioscience and Biotechnology (http://extract.pdrc.re.kr). The 3T3-L1 pre-adipocyte and mouse MC3T3-E1 cells were purchased from the ECACC cell line (Sigma Aldrich, USA). The MC3T3-E1 cells were cultured in Dulbecco’s modified Eagle’s (DMEM) medium and 3T3-L1 cells were cultured in high-DMEM medium, supplemented with 10% FBS, penicillin (100 U/mL) and streptomycin (100 μg/mL). The MC3T3-E1 differentiation was induced by 50 μg/ mL of ascorbic acid and 10 mM of β-glycerophosphate. The 3T3-L1 differentiation was induced by 0.5 mM 3-isobutyl-1-methylxanthine, 1 mm dexamethasone and 1 µg/ mL insulin (MDI). After 3 days of differentiation, the cells were incubated with µg/ mL insulin for 5 days. COV434 cells and 3T3-L1 pre-adipocytes were purchased from the ECACC cell line (Sigma Aldrich, USA). COV434 cells were cultured in Dulbecco’s modified Eagle’s medium (DMEM) with 2 mM glutamine, and 3T3-L1 cells were cultured in high-DMEM, supplemented with 10% FBS, 100 U/mL penicillin, and 100 μg/mL streptomycin. For estradiol production, COV434 cells were treated with 10 µM androstenedione (ADD) for 48 h, and an aliquot of ADD-treated medium was stored at −70 °C until the estradiol assay. The medium was changed every 3 days. All cultured cells were incubated in a humidified atmosphere at 37 °C and at 5% CO_2._

### 4.2. Cell Viability Assay

The 3T3-L1 preadipocytes and MC3T3-E1 cells were incubated in a 96-well plate overnight and treated with single extract of CO and RF and CO + RF mixture in the medium for 48 h. Cell viability was measured by WST assay. Cells were incubated with WST solution (20 µL, 5 mg/mL in phosphate-buffered saline, PBS) for 4 h and absorbances were measured at 450 and 655 nm using a microplate reader (BioTek, Winooski, USA).

### 4.3. Oil Red O Staining

Lipid accumulation in DMI-induced 3T3-L1 cells was measured by oil red O staining. Differentiated cells were fixed with 4% paraformaldehyde for 15 min and washed with PBS three times. Fixed cells were stained with oil red O dye and positive oil red O staining were visualized under a microscope.

### 4.4. Alkaline Phosphatase (ALP) Assay and Mineralized Nodule Formation

After osteoblast differentiation, ALP activity was analyzed using cell lysates in a buffer containing 5 mmol/L p-nitrophenylphosphate, 0.5% Triton X-100, 10 mmol/L Mg^2+^, and 1 mmol/L Tris–HCl (pH 8.8) at 4 °C. Absorbance values of ALP activity were measured at 405 nm by using a microplate reader (BioTek, Winooski, USA). For ALP staining, differentiated osteoblastic cells were fixed in cold 4% paraformaldehyde and stained with a BCIP/NBT (Sigma-Aldrich; St. Louis, MO, USA). Mineralized nodule formation in osteoblastic MC3T3-E1 cells was cultured with induction medium for 21 days. Mineralized cells were fixed in cold 4% paraformaldehyde and calcium deposits of the osteoblast cells were stained with Alizarin red S (Sigma-Aldrich; St. Louis, MO, USA). Positive stained cells were visualized using a light microscope.

### 4.5. Quantitative Reverse-Transcription PCR (qRT-PCR)

Total RNA was extracted using TRIzol reagent (Invitrogen, Carlsbad, USA), according to the manufacturer′s instructions (Beckman Coulter, Brea, USA). The extracted RNA was reverse transcribed with a cDNA Synthesis Kit of RevertAid™ H Minus First Strand (Fermentas Inc., Hanover, USA). Real-time RT-PCR measurements were analyzed by the ABI Prism 7000 Sequence Detection System (Applied Biosystems, Foster City, USA). All PCR amplifications were performed for 40 cycles containing with 150 ng of cDNA using an SYBR Green I qPCR kit (TaKaRa, Shiga, Japan). The specific primers were as follows: 5′-CAA GCA CCT CTG ACA AGG TTC-3′ and 5′-GTT GGC GGC ATA TTC TGC TG-3′ for mouse *Plin1*, 5′-TGT TCC TCT TAA TCC TGC CCA-3′ and 5′-CCA ACC TGC ACA AGT TCC CTT-3′ for mouse *Adipoq*, 5′-TCC CAC GTT TTC ACA TTC GG-3′ and 5′-GGC CAT CCT ATA TGG TAA CGG G-3′ for mouse *Alpl*, 5′-TAAAGTGACAGTGGACGG TCCC-3′ and 5′-CCTCAGTGATTTAGGGCGCA-3′ for mouse *Runx2*, 5′-TAG TGA ACA GAC TCC GGC GCT A-3′ and 5′-ATG GCT TGA AGA CCG CCT ACA-3′ for mouse *Bglap*, 5′- CTT GGA AGG CCG AAA TGA AAT G-3′ and 5′- GGC AGG GCT ATT CTT CTT AGT G-3′ for mouse *ESR1,* 5′- CTG GTC CTG TGA AGG ATG TAA G-3′ and 5′- TTA CGC CGG TTC TTG TCT ATG-3′ for mouse *ESR2*, 5′-TGA CCA CAG TCC ATG CCA TC-3′ and 5′-GAC GGA CAC ATT GGG GGT AG-3′ for mouse *Gapdh,* 5′- GGC TAG GTC ATC CAA AGA GAA G-3′ and 5′- TCT GCA AGG TTA CTA GGG AAA TC-3′ for human ESR1, 5′- TCG CTG CCC TAT GAC GAC A-3′ and 5′- CTT CTT GTA CCT GCG TAC CAG-3′ for human ESR2, and 5′-TGT TGC CAT CAA TGA CCC CTT-3′ and 5′-CTC CAC GAC GTA CTC AGC G-3′ for human GAPDH. By normalizing the levels to those of Gapdh, relative quantification of gene expression was performed using the comparative threshold (Ct) method, as described by the manufacturer (Applied Biosystems). 

### 4.6. Measurement of Estradiol Production

For estradiol quantification, COV434 cells were co-treated with the extract and 10 µM androstenedione (ADD) for 48 h at 37 °C in a 5% CO_2_ atmosphere. The medium was collected and centrifuged to obtain the supernatant. The estradiol levels in the supernatants were quantified using the Homogeneous Time Resolved Fluorescence (HTRF^®^) Estradiol kit (Cisbio, USA) with the TriStar2S LB 942 multimode reader (Berthold Technologies, USA), according to the manufacturer’s instructions.

### 4.7. Ovariectomized Model

Ovariectomized (OVX, *n*= 24) and sham-operated (Sham, *n* = 8) 8-weeks-old female ddY mice were purchased from Shizuoka Laboratory Center Inc. (Hamamatsu, Japan). Mice were maintained on a diet (5.0 g/day) of Formula-M07 (Feedlab Co., Ltd., Hanam, Korea) and tap water (15 mL/day). All mice were housed individually in clear plastic cages under controlled temperature (23 ± 2 °C), humidity (55 ± 5%), and illumination (12-h light/dark cycle). Mice were treated with combined CO + RF extract (75 (L), 150 (M), or 300 (H) mg/kg/day) for 12 weeks. Committee of the Ajou University School of Medicine, and all experiments were conducted in accordance with the institutional guidelines established by the Committee (2016-0062). 

### 4.8. Measurement of Bone Loss in OVX Mice 

After anesthetization using tiletamine/zolazepam (Zoletil; Virbac Laboratories, Carros, France), the mice were placed on the specimen tray for measurements. All mice were placed carefully in the same position. Right femur BMD and % fat were measured using on-board PIXI-mus software for small animals (GE Lunar, Madison, USA). Transverse micro-CT images of the right femur sample were scanned using a micro-CT in the GSTEP (INVEON, SIEMENS, Germany) with a voltage of 60 kV, a current of 400 µA, an exposure time of 400 ms, a magnification of M-H, a CCD readout of 1280 × 1280 and a rotation steps of 360. Reconstruction was performed using Inveon Research Workplace and COBRA_Exxim (SIEMENS, Germany). 

### 4.9. Hematoxylin and Eosin (H&E) Staining 

The animals were euthanized immediately before abdominal visceral fat, liver, and uterine tissue collection. Tissues were collected into cold 4% paraformaldehyde in 0.1 M phosphate buffer at pH 7.4 and post fixed overnight at 4 °C in the same solution. Each bone sample was cut into 3 μm coronal planes and embedded in paraffin for tissue sectioning. The sections were stained with hematoxylin and eosin (H&E) to evaluate the changes in fat, liver, and uterine tissues.

### 4.10. Blood Sampling and Serum Leptin and Insulin

Blood samples were collected from the left ventricle using heparinized syringes. Serum samples were separated by centrifugation at 1200× *g* at 4 °C for 15 min and measured for adipogenic makers (leptin and insulin) using multiplex assays analyzed with Luminex (Merck Millipore, Burlington, MA, USA) following the manufacturer’s instructions.

### 4.11. Statistical Analysis 

A statistical software package (SPSS 11.0 for Windows, SPSS Inc., Chicago, IL, USA) was used for performing the statistical tests. The statistical significance of differences was assessed by the Student′s *t*-test. Results were considered significant at a value of *p* < 0.05. The results were expressed as mean ± SEM.

## 5. Conclusions

This study demonstrated that combination of CO + RF at a 7:3 ratio had anti-obesity and anti-osteoporotic effects and enhanced the estrogenic activity in preadipocyte 3T3-L1, preosteoblast MC3T3-E1 cells, and COV434 granulosa cells in vitro and in vivo. Thus, a combination of CO and RF extracts may be a potential candidate for the treatment and prevention of women’s post-menopausal symptoms.

## Supplementary Materials

The following are available online at https://www.mdpi.com/2072-6643/12/2/369/s1, Figure S1: Effect of CO and RF extract on cell proliferation in preadipocyte 3T3-L1 cells. Cells were treated with CO or RF extract or their combination at a 7:3 ratio (50 μg/mL) for 48 h and the cell viability was assessed by WST, Figure S2: Effect of CO and RF extract on cell proliferation in preadipocyte preosteoblast 3T3-L1 cells. Cells were treated with CO or RF extract or their combination at a 7:3 ratio (50 μg/mL) for 48 h and the cell viability was assessed by WST, Figure S3: Effects of CO + RF extracts on mRNA expression of ESR2 in androstenedione (ADD)-induced COV434 granulosa cells. (**A**) Stimulation of estradiol production by treatment of 10 µM ADD in COV434 cells for 48h. Estradiol level in the supernatant of the cell culture medium was measured by ELISA kit. (**B**) COV434 cells were co-treated with CO or RF extract or their combination at a 7:3 ratio (50 μg/mL) and 10 µM ADD for 48h, and the mRNA expression level of ESR2 gene was calculated quantitatively by qRT-PCR, Figure S4: Effects of CO + RF extracts on mRNA expression of ESR1 (**A**) and ESR2 (**B**) genes in preosteoblastic MC3T3-E1 cells. Cells were treated with CO or RF extract or their combination (6:4, 7:3, and 8:2 ratios; 50 µg/mL). The relative mRNA levels of ESR1 and ESR2 genes were analyzed by RT-PCR. *: *p* < 0.05 vs. Control.
