# Supplementary material for: Anti-Menopausal Effects of Cornus officinalis and Ribes fasciculatum Extract In Vitro and In Vivo
Source: Nutrients. 2020 Jan 30;12(2):369. doi: 10.3390/nu12020369 (PMC7071277; doi:10.3390/nu12020369)
Supplement: Supplementary file 1 [file nutrients-12-00369-s001.pdf]

Supplementary Figures

Anti-menopausal effects of *Cornus officinalis* and *Ribes fasciculatum* extract in vitro and in vivo

Eunkuk Park[1,2], Eunguk Lim[1,2], Subin Yeo[3], Yoonjoong Yong[3], Junga Yang[3], and Seon-Yong Jeong[1,2,3]

[1] Department of Medical Genetics, Ajou University School of Medicine, Suwon, Republic of Korea
[2] Department of Biomedical Sciences, Ajou University Graduate School of Medicine, Suwon, Republic of Korea
[3] Nine B Co. Ltd., Daejeon 34121, Republic of Korea

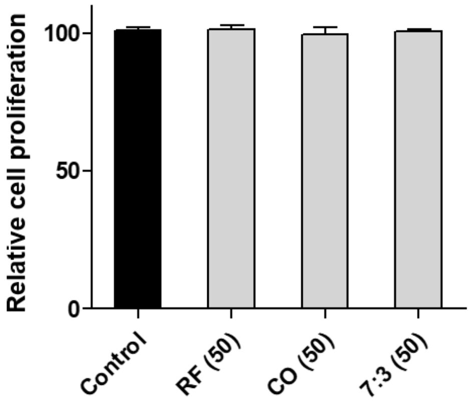

Supplementary Figure S1. Effect of CO and RF extract on cell proliferation in preadipocyte 3T3-L1 cells. Cells were treated with CO or RF extract or their combination at a 7:3 ratio (50 μg/mL) for 48 h and the cell viability was assessed by WST.

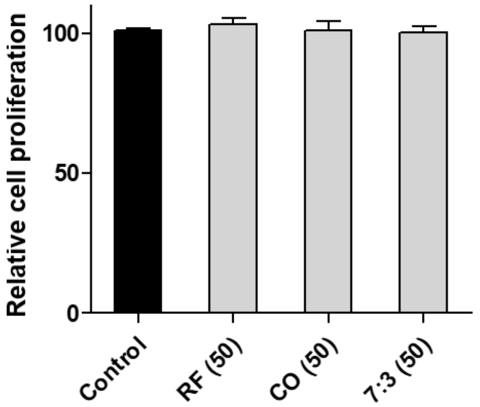

Supplementary Figure S2. Effect of CO and RF extract on cell proliferation in preadipocyte preosteoblast 3T3-L1 cells. Cells were treated with CO or RF extract or their combination at a 7:3 ratio (50 μg/mL) for 48 h and the cell viability was assessed by WST.

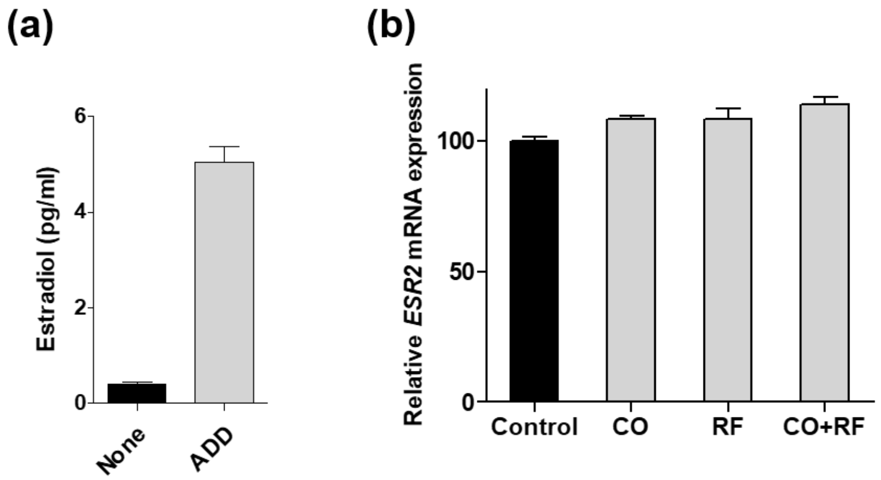

Supplementary Figure S3. Effects of CO+RF extracts on mRNA expression of *ESR2* in androstenedione (ADD)-induced COV434 granulosa cells. (**A**) Stimulation of estradiol production by treatment of 10 μM ADD in COV434 cells for 48h. Estradiol level in the supernatant of the cell culture medium was measured by ELISA kit. (**B**) COV434 cells were co-treated with CO or RF extract or their combination at a 7:3 ratio (50 μg/mL) and 10 μM ADD for 48h, and the mRNA expression level of ESR2 gene was calculated quantitatively by qRT-PCR.

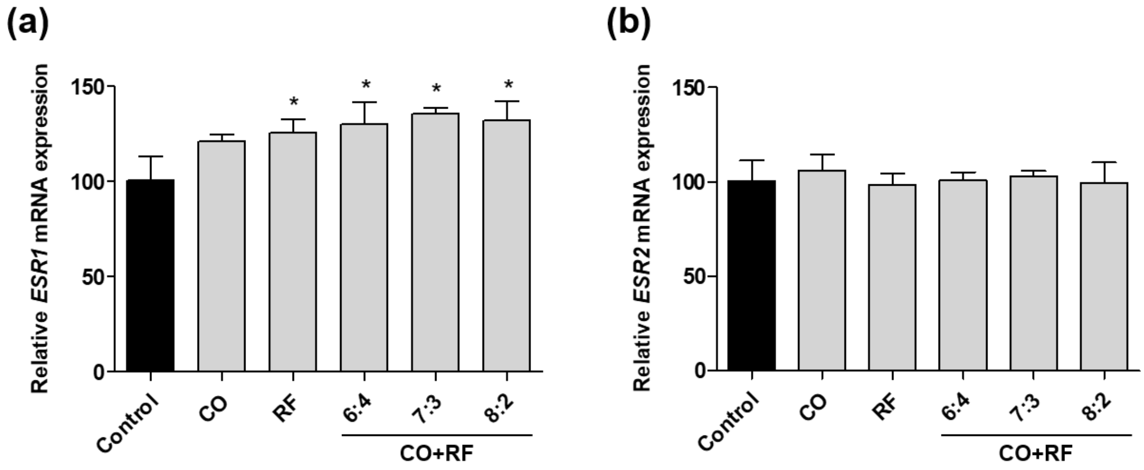

Supplementary Figure S4. Effects of CO+RF extracts on mRNA expression of *ESR1* (**A**) and *ESR2* (**B**) genes in preosteoblastic MC3T3-E1 cells. Cells were treated with CO or RF extract or their combination (6:4, 7:3, and 8:2 ratios; 50 μg/ml). The relative mRNA levels of *ESR1* and *ESR2* genes were analyzed by RT-PCR. *: $p < 0.05$ vs. Control.
